# Supplementary material for: Measuring ligand efficacy at the mu-opioid receptor using a conformational biosensor
Source: eLife. 2018 Jun 22;7:e32499. doi: 10.7554/eLife.32499 (PMC6042960; doi:10.7554/eLife.32499)
Supplement: Figure 3—source data 1. — Ligand number refers to the list in legend to Figure 3; the t1/2 for association of NB39 is from Table 1; Maximum stimulation of [35S]GTPγS binding by each ligand is taken from Livingston and Traynor (2014); Ehlert’s efficacy value (e, Ehlert, 1985) determined for each ligand using [35S]GTPγS assay data and ligand affinities, taken from Livingston and Traynor (2014); Shift in ligand affinity in the presence of 100 mM NaCl and 10 μM GTPγS, from Livingston and Traynor (2014); Efficacy (τ) values for stimulation of [35S]GTPγS binding (Livingston and Traynor, 2014) calculated according to Black and Leff (1983); Efficacy (τ) values for arrestin recruitment taken from McPherson et al. (2010). [file elife-32499-fig3-data1.docx]

| **Figure 3- Source Data** | | |  |  |  |  |
| --- | --- | --- | --- | --- | --- | --- |
|  |  |  |  |  |  |  |
| **Drug #** | **t1/2 (sec)** | **G Protein (Max)** | **Ehlert, e** | **Log Affinity Shift Na+/GTPgS** | **Log(τ) [GTPγS]** | **Log(τ) [arrestin]** |
| 1 | 3.5 | 100 | 4.7 |  | n/a | n/a |
| 2 | 3.9 | 100 | 2.1 | 1.34 | n/a | 0.86 |
| 3 | 7.1 | 100 | 3.6 | 1.25 | n/a | 0.56 |
| 4 | 3.6 | 100 | 1.1 | 1.35 | n/a | n/a |
| 5 | 8.5 | 61 | 0.5 | 1.2 | 0.24 | 0.22 |
| 6 | 29.7 | 5 | n/a | 1 | -1.39 | n/a |
| 7 | 6.9 | 94 | 1.2 | 1.25 | n/a | 0.84 |
| 8 | 3.2 | 86 | 2.9 | 1.31 | n/a | n/a |
| 9 | 15.9 | 57 | n/a | n/a | 0.12 | 0.23 |
| 10 | 3.9 | 100 | 4.7 | n/a | n/a | 0.8 |
| 11 | 9.2 | 61 | 0.5 | 1.23 | 0.23 | 0.64 |
| 12 | 5.3 | 100 | 1.8 | 1.3 | n/a | 0.86 |
| 13 | 16.7 | 58 | n/a | n/a | 0.11 | n/a |
| 14 | 11.9 | 21 | 0.2 | 1.02 | -0.626 | n/a |
